# COVID-19 advanced cardiovascular and clinical phenotyping dataset

**DOI:** 10.1016/j.dib.2026.113139

**Published:** 2026-08-04

**Authors:** Oscar E. Barreras, Daniel Cuevas-González, Marco A. Reyna, Edgar García-Duarte, Juan Pablo García-Vázquez, Juan C. Delgado-Torres, Martin Aaron Sánchez-Barajas, Roberto L. Avitia

**Affiliations:** a Instituto de Ingeniería—Universidad Autónoma de Baja California, Mexicali 21280, Mexico; b Cuerpo Académico de Bioingeniería y Salud Ambiental— Universidad Autónoma de Baja California, Mexicali 21280, Mexico; c Hospital General de Zona 30—Instituto Mexicano del Seguro Social, Mexicali 21100, Mexico

**Keywords:** SARS-CoV-2, Aftereffects of SARS-CoV-2, Ventricular late potentials, Risk stratification, Cardiac electrophysiology, Clinical biomarkers, Cardiovascular variables

## Abstract

This article describes a structured clinical dataset collected from 100 adult patients who were previously hospitalized due to moderate-to-severe COVID-19 infection. Data were obtained prospectively at a second-level care hospital in Mexicali, Baja California, Mexico. The dataset includes demographic characteristics, anthropometric measurements, traditional cardiovascular risk factors, general blood test, Questionnaire-derived variables, 12-lead electrocardiographic (ECG) parameters, signal-averaged electrocardiogram (SAECG) measurements, and heart rate variability (HRV) metrics. Questionnaire-derived variables include medical history, cardiovascular risk factors, medication use, lifestyle-related factors, and COVID-19 vaccination information. Laboratory variables include metabolic, renal, lipid, electrolyte, and hematological parameters obtained from routine clinical blood analyses. The 12-lead ECG data include cardiac rhythm classification, measurements of wave and interval durations, electrical axis parameters, and precordial voltage amplitudes. SAECG variables are provided as raw numerical measurements, including total QRS duration, low-amplitude signal duration below 40 µV (LAS40), and root mean square voltage of the last 40 ms (RMS40), together with a variable indicating the presence or absence of ventricular late potentials. HRV parameters are available as continuous time-domain and frequency-domain values, allowing future researchers to apply different clinical thresholds or computational approaches. The dataset is organized into multiple structured Comma-Separated Values (CSV) files distributed across dedicated subdirectories within the Mendeley Data repository. The repository also includes supporting PDF documentation, such as the ethics approval document containing the protocol number, the questionnaire applied during data collection, and the informed consent form. An overview of each variable is provided, and only anonymized patient identifiers are included; no personally identifiable information is contained in the dataset. These data may support future exploratory studies on cardiovascular alterations in post-COVID populations and machine learning applications. The structured format is intended to facilitate secondary data analyses, such as correlational studies and the identification of associations between clinical and electrocardiographic variables.

Specifications Table

**Table:** 

Subject	Cardiology and Cardiovascular Medicine.
Specific subject area	Cardiovascular risk factors, 12-lead ECG, SAECG and HRV variables in post-COVID adults.
Type of data	Comma-Separated Values (CSV) files and Portable Document Format (PDF) files
Data collection	The data were collected from 100 adults previously hospitalized for moderate-to-severe COVID-19 at Hospital General de Zona No 30, Instituto Mexicano del Seguro Social (IMSS) and Healthcare System, Mexicali. Blood pressure was measured using Omron HEM-7121 and Mindray uMEC10 monitors. 12-lead ECG, HRV, and SAECG were recorded with an EDAN SE-1202 system. Laboratory analyses were performed in a certified hospital laboratory. Inclusion/exclusion criteria followed approved IMSS protocol.
Data source location	Hospital General Zona No 30, IMSS (32°39′39.9″N, 115°27′59.7″W).
Data accessibility	Repository name: Mendeley Data Data identification number: 10.17632/hxcnkf62bh.1 Direct URL to data: https://data.mendeley.com/datasets/hxcnkf62bh/1
Related research article	None.

## 1 Value of the Data

• This dataset provides a structured combination of traditional cardiovascular risk factors (demographic and binary classifications), laboratory variables include metabolic, renal, lipid, electrolyte, and hematological parameters obtained from routine clinical blood analyses, and raw numerical values for standard 12-lead ECG, SAECG and HRV parameters.
• The dataset will allow researchers to apply different analytical thresholds, statistical techniques, or computational models according to their own methodological approaches.
• Researchers in cardiology, biomedical engineering, epidemiology, and data science may reuse these data to explore associations between clinical markers and electrocardiographic variables in post-COVID populations. The structured format supports secondary analyses, such as multivariate analysis, multivariate correlation studies, and machine learning applications.
• The dataset may serve as a reference for future studies exploring cardiovascular alterations in post-COVID populations, particularly in middle-income healthcare settings. It is organized into multiple subdirectories, each containing structured CSV files for the respective categories, to facilitates reproducibility and secondary data analysis.

## 2 Background

Cardiovascular diseases (CVDs) remain the leading cause of mortality worldwide [1]. According to the World Health Organization, an estimated 19.8 million people died from CVDs in 2022 [1]. Traditional cardiovascular risk assessment is commonly based on demographic characteristics, clinical history, and both modifiable and non-modifiable cardiovascular risk factors [2]. However, electrocardiographic markers such as fragmented QRS (fQRS), ventricular late potentials detected by SAECG, and HRV have been associated with arrhythmic risk and adverse cardiovascular outcomes in different clinical settings [[3], [4], [5]].

During the COVID-19 pandemic, increasing evidence suggested potential cardiovascular involvement both during the acute phase and in the post-infection period [[6], [7], [8]]. Some studies reported associations between electrocardiographic alterations and adverse outcomes in patients with SARS-CoV-2 infection [[9], [10], [11], [12]]. Despite this, datasets combining traditional cardiovascular risk factors, blood chemistry, standard 12-lead ECG, ventricular late potentials and HRV measurements in post-COVID adult populations remain limited.

This dataset was compiled to provide structured clinical and electrocardiographic variables collected from adults previously hospitalized due to moderate-to-severe COVID-19. The data are organized to facilitate secondary analyses and future predictive modelling studies.

## 3 Data Description

The structure of the dataset is summarized in Table 1. All data are de-identified, meaning that no personally identifiable information, such as name, address, phone number, or date of birth, is included. Each patient is identified by an anonymized numerical code (Patient_ID).

**Table 1:** Content of “Dataset of cardiovascular risk and electrocardiographic variables in adults post moderate-to-severe COVID-19″ dataset from Mendeley Data Repository.

Subdirectory title	Variables
Protocol information	• PDF file of Approval document with protocol number (Spanish only). • PDF file of applied questionnaire in English. • PDF file of the informed consent form in English.
Demographics_and_Clinical	Patient_ID, Registration_date, Age (years), Weight_kg (kg), Height_cm (cm), Sex (Male/Female), Systolic_blood_pressure (mm Hg), Diastolic_blood_pressure (mm Hg), CVD_risk_percent (%).
Questionnaire	ID, Registration_date, Yes/No (1/0) questions: Received_1st_dose, Received_2nd_dose, Received_3rd_dose, COVID_before_vaccination, Hypertension, Hypertension_medication, Diabetes, Cholesterol_medication, Loss_of_consciousness, Frequent_alcohol_use, Aspirin_treatment, Tobacco_use, Family_history_cardiovascular_disease; Vaccine type (category): 1st_dose, 2nd_dose, 3rd_dose; Vaccination_schedule (category); Year_last_vaccine (year).
Laboratory_Results	Metabolic and renal profile: Glucose (mg/dL), Urea (mg/dL), BUN (mg/dL), Creatinine (mg/dL), Albumin (g/dL); Lipid profile: Total_cholesterol (mg/dL), Triglycerides (mg/dL), HDL (mg/dL), LDL (mg/dL), VLDL (mg/dL); Serum electrolytes and minerals: Sodium (mg/dL), Potassium (mEq/L), Chloride (mEq/L), Calcium (mg/dL), Phosphorus (mg/dL), Magnesium (mEq/day); Complete blood count with differential: Leukocytes (10³/µL), Hemoglobin (g/dL), Hematocrit (%), Erythrocytes (10⁶ cells/µL), Platelets (10³/µL), MCH (pg), MCHC (g/dL), MPV (fL), RDW (%), MCV (fL), Lymphocytes_percent (%), Neutrophils_percent (%), Monocytes_percent (%), Eosinophils_percent (%), Basophils_percent (%), Lymphocytes_count (10³/µL), Neutrophils_count (10³/µL), Monocytes_count (10³/µL), Eosinophils_count (10³/µL), Basophils_count (10³/µL).
Standard_ECG	12-lead ECG conduction and waveform duration parameters: P_ms (ms), PR_ms (ms), QRS_ms (ms), QT_ms (ms), QTcBz_ms (ms); Electrical axis measurements: P_axis_deg (degrees), QRS_axis_deg (degrees), T_axis_deg (degrees); Precordial voltage amplitudes: RV5_mV (mV), SV1_mV (mV); Cardiac rhythm classification: Rhythm (category).
Signal_Average_ECG	Recording metadata (Registration_date, Registration_time); Signal averaging characteristics: Average_beat_duration_s (s), Averaged_beats (beats); QRS duration parameters: Standard_QRS_ms (ms), Total_QRS_ms (ms); Low-amplitude signal parameters: LAS40_ms (ms), RMS40_uV (µV); Ventricular late potential classification: VLP_presence (1 = presence, 0 = absence); Signal quality indicators: Noise_uV (µV), Filter (Hz).
Heart_Rate_Variability	Recording metadata (Registration_date, Registration_time, Measurement_time); Beat count and basic RR interval metrics: Total_beats (beats), HR (bpm), Mean_RR_interval (ms), Max_RR_interval (ms), Min_RR_interval (ms), Max_Min_ratio (unitless); Time-domain parameters: SDNN (ms), SDSD (ms), RMSSD (ms), NN50 (count), PNN50 (%); Geometric parameters: TINN (ms), Triangular_index (unitless); Frequency-domain parameters: LF (ms²), HF (ms²), Total_power (ms²); Normalized frequency components: LF_normalized (unitless), HF_normalized (unitless); Sympathovagal balance index: LF_HF_ratio (unitless).

The main directory has seven subdirectories, one containing 3 PDF files with protocol information and six containing a CSV file corresponding to a specific category of variables collected and a XLSX file with an overview of the dataset, which provides a description of all variables included in the dataset. It contains the operational definitions of the variables, including the name, a description, unit of measurement, and data type. Table 1 summarizes the structure and contents of each folder.

The Demographics_and_Clinical file contains baseline demographic and anthropometric information recorded during clinical evaluation. Variables include anonymized patient identifier (ID), registration date, age (years), weight (kg), height (cm), sex, and resting blood pressure measurements (systolic and diastolic blood pressure, mmHg).

This file also includes the 10-year atherosclerotic cardiovascular disease (ASCVD) risk percentage, calculated with the validated ACC/AHA ASCVD Risk Estimator Plus. The risk estimation was based on clinical inputs including age, sex, total and HDL cholesterol, systolic blood pressure, smoking status, and history of diabetes or antihypertensive medication use. This assessment was applied to participants aged 40–79 years, consistent with the tool’s validated population; for participants outside this age range or with missing clinical inputs required for the model, the risk value was recorded as empty (null).

The Questionnaire file contains dichotomous variables derived from a structured patient questionnaire administered during evaluation. These variables include modifiable cardiovascular risk factors (e.g., tobacco use, hypertension, diabetes), medication use, alcohol consumption, history of loss of consciousness, family history of cardiovascular disease, and COVID-19 vaccination-related information, including type of vaccine received and vaccination schedule. Binary responses are coded as categorical variables, “1″ for a yes and “0” for a no.

The Laboratory_Results file includes biochemical and hematological parameters obtained from blood samples analyzed at the institutional clinical laboratory of the IMSS. All parameters are presented using the original nomenclature and measurement units as documented in the institutional records. Variables include metabolic and renal markers, lipid profile, serum electrolytes and minerals, and complete blood count with differential.

The Standard_ECG file contains parameters automatically extracted from 12-lead electrocardiographic recordings acquired using the EDAN SE-1202 system. Variables include heart rate (HR_bpm), conduction and waveform duration parameters (P_ms, PR_ms, QRS_ms, QT_ms, QTcBz_ms), electrical axis measurements (P_axis_deg, QRS_axis_deg, T_axis_deg), precordial voltage amplitudes (RV5_mV, SV1_mV), and rhythm classification.

The Signal_Average_ECG file provides signal-averaged electrocardiographic measurements acquired using the EDAN SE-1202 system. Variables of recording data (registration date and time), signal averaging characteristics (average beat duration and number of averaged beats), QRS duration parameters (standard and total filtered QRS duration), low-amplitude signal parameters (LAS40_ms and RMS40_uV), ventricular late potential classification (VLP_presence, 1 for a presence and 0 for absence), and signal quality indicators (noise level and applied filter).

The Heart_Rate_Variability file contains heart rate variability parameters derived from ECG recordings using the EDAN SE-1202 system. Variables include recording metadata, total beats analyzed, basic RR interval metrics (mean, maximum, minimum, and ratio), time-domain parameters (SDNN, SDSD, RMSSD, NN50, pNN50), geometric parameters (TINN, triangular index), frequency-domain parameters (LF, HF, total power), normalized components (LF_normalized, HF_normalized), and the LF/HF ratio.

## 4 Experimental Design, Materials and Methods

A research protocol was developed and approved in collaboration with the IMSS to establish this clinical dataset. The study was formally reviewed and approved by the corresponding Institutional Research and Ethics Committee (approval number: R-2024–205–097). Additional information regarding the ethical approval is available in the data repository, as described in the specification table. Participants were randomly selected from an institutional database of 4130 patients with confirmed SARS-CoV-2 infection (via RT-PCR) who were hospitalized due to moderate-to-severe COVID-19. Severity was determined upon hospital admission based on clinical assessment of symptomatic presentation, respiratory distress, and oxygen saturation levels.

**Fig. 1:**
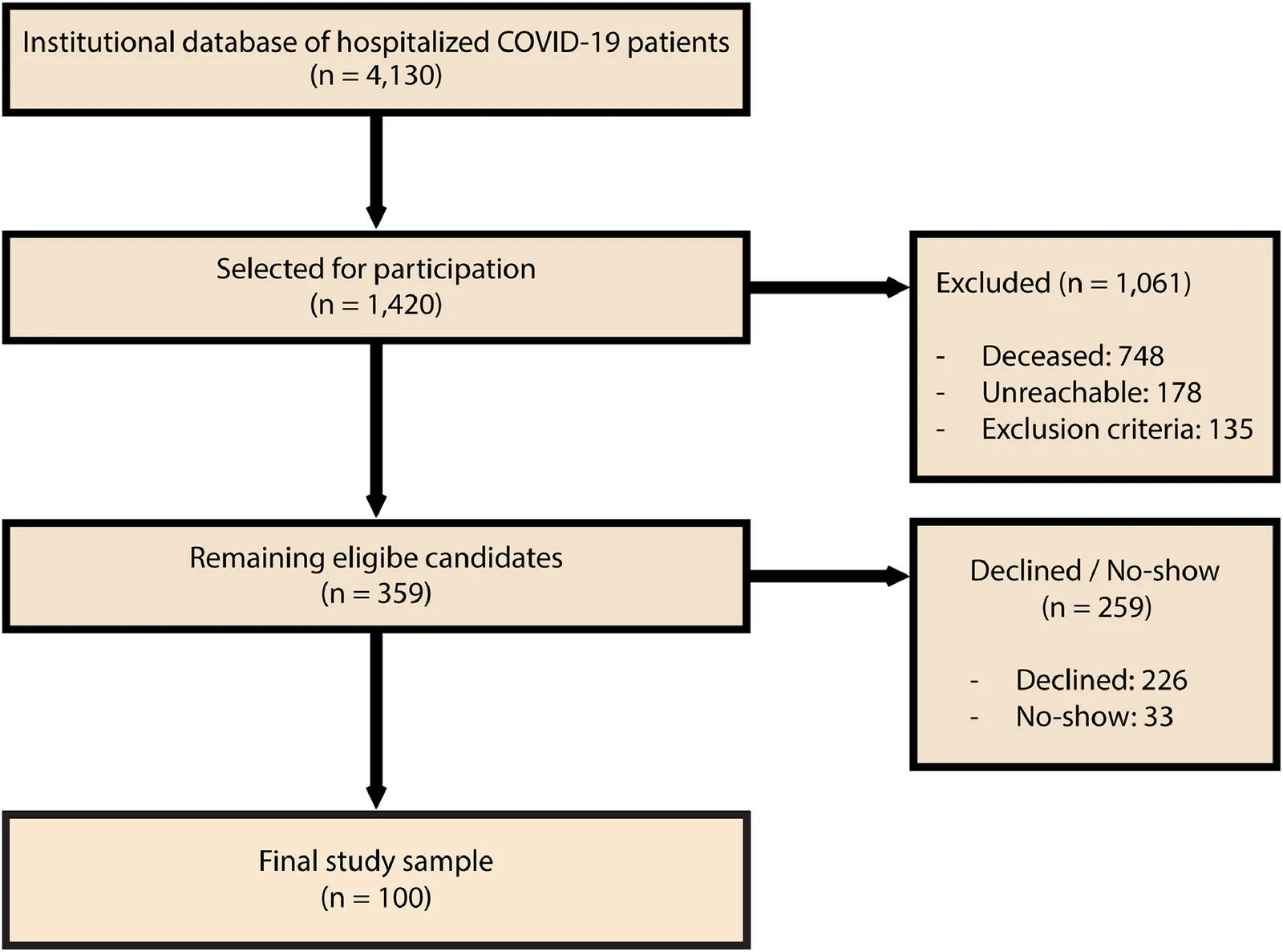
Participant recruitment and selection flow diagram.

A total of 1420 individuals were contacted by telephone; 748 were deceased, 178 were unreachable, and 135 did not meet eligibility criteria (age >80, n = 120; history of cardiovascular disease, n = 10; or lack of social security coverage, n = 5). Of the remaining 359 candidates, 226 declined participation and 33 failed to attend their scheduled evaluation, resulting in a final sample of 100 participants. Fig. 1 shows a flow diagram of the participant recruitment and selection process.

During the initial contact, the study objectives were explained and exclusion criteria were assessed according to the approved protocol, including prior history of myocardial infarction, presence of cardiac implantable electronic devices such as pacemakers, and other predefined cardiovascular conditions. The patients who met eligibility criteria and agreed to participate were scheduled for an in-person evaluation at the Specialty Tower facilities of Hospital General de Zona No 30 in Mexicali, Baja California, Mexico.

Participants were instructed to attend the evaluation in fasting conditions to allow blood sample collection. Morning medications were permitted unless contraindicated due to fasting requirements, such as medications requiring food intake. Upon arrival, beginning at 7:00 a.m., participants were seated and provided with a detailed explanation of the procedures, risks, and benefits of the study. Written informed consent was obtained in accordance with institutional research guidelines from the Health Research Coordination Unit.

After a resting period of approximately 5–10 min, blood pressure was measured under standardized resting conditions. For participants numbered 001–050, blood pressure was recorded using an Omron HEM-7121 automated monitor, whereas participants 051–100 were evaluated using a Mindray uMEC10 monitor. Blood samples (approximately 8 mL) were collected into specific vacutainer tubes based on the required laboratory analysis using sterile, single-use hypodermic syringes. A 4 ml sample was collected in a K2-EDTA (purple-top) tube for hematological analysis (CBC). A separate 4 ml sample was collected in a serum-separating (yellow-top) tube containing a clot activator and gel for biochemical and electrolyte analyses. Anthropometric measurements, including weight and height, were obtained using a calibrated scale with stadiometer. Blood pressure measurement, blood sampling, and anthropometric assessment were performed by a licensed registered nurse.

A structured questionnaire was administered by the doctor responsible for the study to collect information on modifiable cardiovascular risk factors, medication use, family history of cardiovascular disease, and COVID-19 vaccination history.

**Fig. 2:**
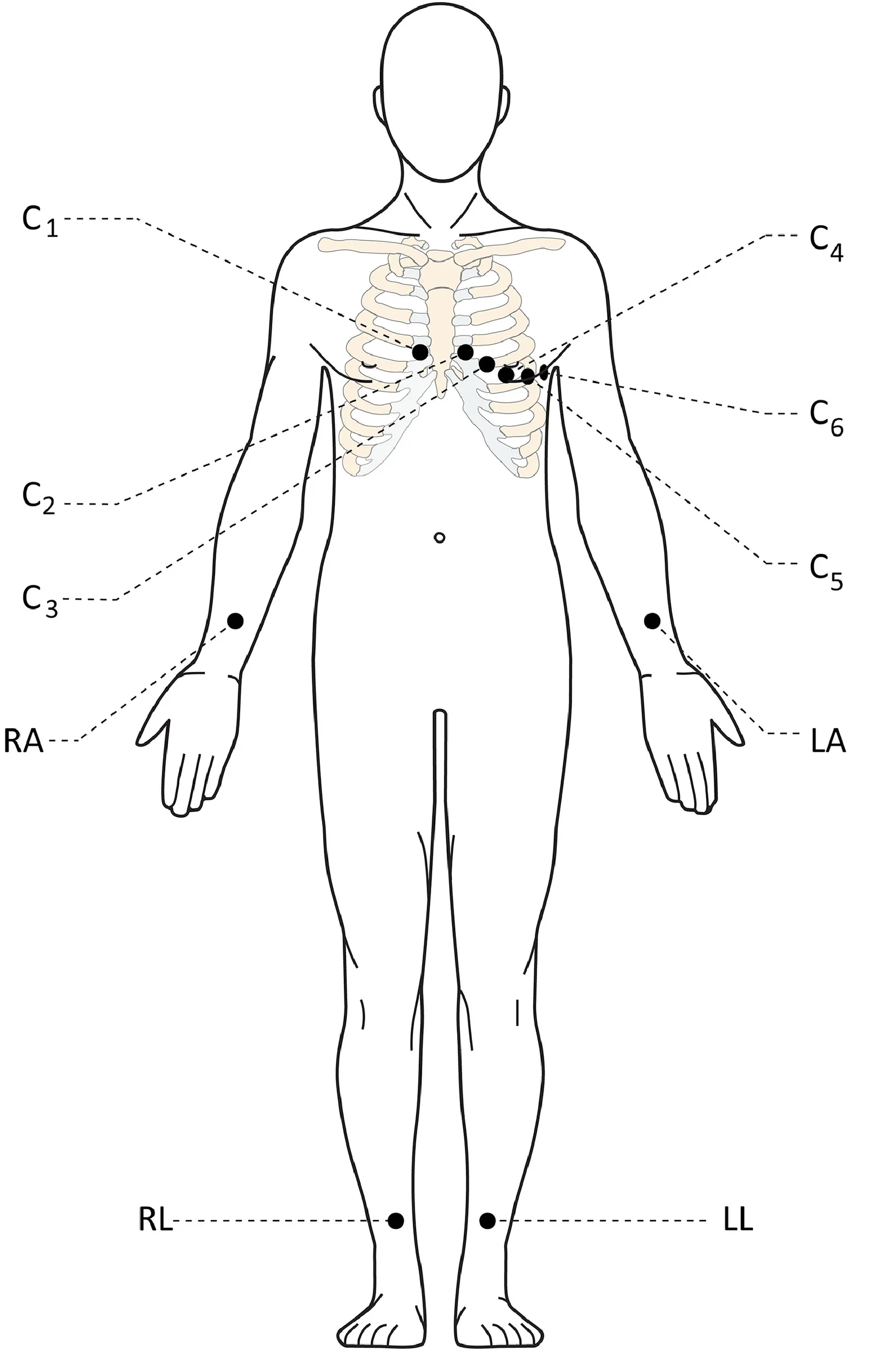
Standard 12-lead electrocardiogram electrode placement. C1–C6 correspond to the precordial (chest) electrodes, while RA, LA, RL, and LL represent the right arm, left arm, right leg, and left leg electrodes, respectively.

For electrocardiographic assessment, participants were positioned in upright sitting posture on an examination table and a standard 12-lead electrode configuration was applied. Recordings were obtained using the EDAN SE-1202 electrocardiograph (24-bit A/D converter, 64,000 Hz sampling frequency) under resting conditions. A 3-minute acquisition was performed for extraction of standard 12-lead ECG parameters. For HRV a 5-minute resting recording was obtained using Lead II from the same electrode placement shown in Fig. 2. Participants were instructed to remain still and breathe normally to minimize motion artifacts and signal noise for accurate R-peak detection by the automated algorithm. HRV indices were extracted as raw, directly from the clinical report generated by the device’s integrated software. No additional external signal processing or filtering was applied. Fig. 2 illustrates the standard 12-lead electrode placement used for ECG and HRV recordings.

**Fig. 3:**
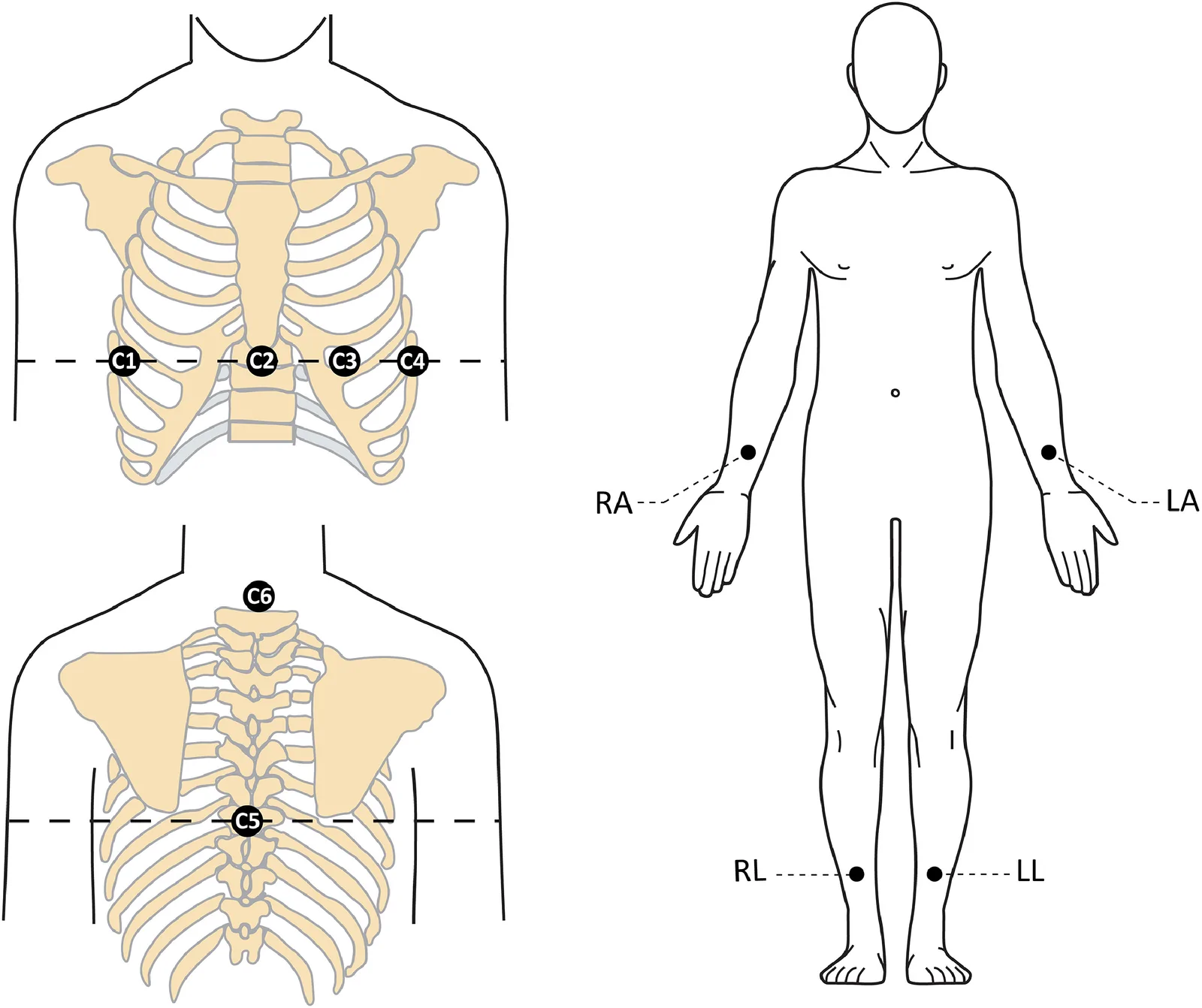
Electrode configuration used for signal-averaged electrocardiography (SAECG) recordings according to the orthogonal Frank lead system. Electrodes C1–C6 correspond to the chest and back positions, while RA, LA, RL, and LL represent the limb electrodes.

For signal-averaged electrocardiography (SAECG), electrodes were repositioned according to the orthogonal Frank lead system configuration. A 5-minute recording was performed to maximize beat averaging and enhance detection of low-amplitude signals. Participants were instructed to remain relaxed and avoid movement during recording. The system automatically calculated filtered total QRS duration, duration of signals below 40 µV (LAS40), root mean square voltage of the last 40 ms (RMS40), along with signal quality indicators. SAECG was considered positive for ventricular late potentials based on standard criteria suggested by the Task Force Committee of the European Society of Cardiology, American Heart Association, and American College of Cardiology [13,14]. A positive result required the presence of at least two of the following abnormalities: total QRS duration > 114 ms, LAS40 > 38 ms, and RMS40 < 20 µV. The orthogonal Frank lead electrode configuration used for SAECG recordings is shown in Fig. 3.

In addition to the clinical and electrocardiographic data files, the Mendeley Data repository includes supporting institutional documentation related to the study, such as the Informed Consent Form, the applied questionnaire and the documentation associated with the ethics committee approval.

All study data were organized into separate subdirectories according to data type. Demographic variables, questionnaire responses, laboratory test results, 12-lead ECG measurements, HRV parameters, and SAECG parameters were stored in independent CSV files within their corresponding subdirectory. This structure was designed to facilitate data management and allow users to access each category of information separately. No statistical transformation, normalization, or imputation procedures were applied prior to dataset compilation. Each participant was assigned an anonymized identification code to ensure confidentiality.

## Limitations

This dataset includes a total of 100 patients from a single healthcare institution in Mexicali, Baja California, Mexico who have recovered from COVID-19; however, we do not have records from before the illness. Blood pressure measurements were obtained using two different automated devices. For participants 001–050, blood pressure was measured using the hospital's Omron HEM-7121, while for participants 051–100, a Mindray uMEC10 monitor was incorporated. However, both devices were clinically validated. In some cases, specific parameters from general blood tests performed by the IMSS laboratory were unavailable, incomplete, or not consistently reported across all patients.

## Ethics Statement

This study involved human participants and was conducted in accordance with the ethical principles outlined in the Declaration of Helsinki. Written informed consent was obtained from all participants prior to data collection.

The research protocol was reviewed and approved by the institutional Research and Ethics Committee of the IMSS. The official approval document, protocol number, and a copy of the informed consent form is available in the data repository associated with this dataset.

All data were anonymized prior to compilation. No personal information is included in the published dataset.

## CRediT Author Statement

**Oscar Eduardo Barreras-Calderon:** Writing- Reviewing and Editing, Investigation, Data curation. **Daniel Cuevas-González:** Writing- Reviewing and Editing, Funding acquisition, Conceptualization, Writing, Supervision. **Marco Antonio Reyna-Carranza:** Visualization, Conceptualization, Investigation. **Edgar García-Duarte:** Writing, Validation, Supervision. **Juan Pablo García-Vázquez:** Writing – review & editing, validation, and formal analysis. **Juan Carlos Delgado-Torres:** Methodology, Writing- Reviewing and Editing. **Martin Aaron Sánchez-Barajas:** Original draft preparation, Data curation. **Roberto López-Avitia:** Methodology, Validation, Software.

## Ethics Declaration

Written informed consent to take part in the study and to publish the article has been obtained from all participants or their legal representatives. The privacy rights of participants have been observed.

This study was performed in compliance with relevant laws, regulatory frameworks and guidelines where the research took place.This study was approved by the Comite Local de Investigación en Salud 205.(Approval No R-2024–205–097).

## Data Availability

Mendeley DataDataset of cardiovascular risk and electrocardiographic variables in adults post moderate-to-severe COVID-19. (Original data).
